# Medical barriers to emergency contraception: a cross-sectional survey of doctors in North India

**DOI:** 10.9745/GHSP-D-13-00139

**Published:** 2014-04-01

**Authors:** M E Khan, Anvita Dixit, Isha Bhatnagar, Martha Brady

**Affiliations:** a Population Council, New Delhi, India; b Population Council, New York, NY, USA

## Abstract

**Background:**

Some medical doctors in India have publicly expressed opposition to making emergency contraceptive pills (ECPs) easily accessible, even though ECPs are included in the method mix of the Ministry of Health and Family Welfare program and as an over-the-counter (OTC) product. Such opposition affects access to ECPs by influencing policy, procurement, and distribution, besides stigmatizing the ECP user. This study was conducted to assess ECP knowledge, attitudes, and practices of doctors in North India.

**Methods:**

A cross-sectional survey of 83 doctors who provide ECPs, randomly selected from 3 cities in the state of Uttar Pradesh, was conducted in 2011. The quantitative data were complemented by 19 in-depth interviews with purposively selected senior gynecologists and other opinion leaders.

**Results:**

All surveyed physicians cited the correct dose and regimen for ECPs. However, the large majority of those surveyed believed that ECPs work by preventing implantation. (The best evidence currently indicates that ECPs do *not* work by preventing implantation.) Most doctors also believed incorrectly that ECPs have several contraindications and side effects. They also had strong reservations against OTC provision of ECPs by pharmacists and community health workers (CHWs) and negative attitudes toward ECP users, which serve as serious medical barriers to mainstreaming use of ECPs.

**Conclusion:**

Physicians and their professional associations exert a strong influence on the operationalization of national contraceptive policies. Evidence-based advocacy and educational campaigns targeting doctors are needed to address and resolve their reservations about ECPs, particularly about its provision as an OTC product and its distribution by CHWs. Partnerships with medical associations can help reduce doctors' negative attitudes and create a conducive environment for influencing clinical practices. Such changes are needed to increase the availability and use of ECPs as part of a package of a full range of contraceptive method options to prevent unwanted pregnancy among the most vulnerable populations.

## Abstract (precis)

Emergency contraceptive pills (ECPs) are extremely safe and do not interfere with implantation. Yet many surveyed physicians in India did not know that there are no contraindications to using ECPs, and many had negative attitudes about ECP users. Most were against having ECPs available over-the-counter and wanted to impose age restrictions. Efforts are needed to address such misconceptions that might lead to limiting ECP availability.

## INTRODUCTION

Despite a long history of family planning programs in India, about 25% of pregnancies and births in the country are unplanned.^1^ In addition, there is continuing high unmet need for family planning, estimated at 21%,^2^ which contributes to short birth intervals and high numbers of abortions (6.7 million annually).^3^ These abortions are often illegal, unsafe, and performed under unhygienic conditions. All these factors lead to high maternal mortality (212 per 100,000 live births) and morbidity.^4^

Emergency contraceptive pills (ECPs) give women a chance to prevent unwanted pregnancy if used within 5 days of unprotected sex or contraceptive failure. The primary mechanism of action is by preventing ovulation (Table 1). ECPs do not affect an established pregnancy, and there are no data supporting interference with implantation of a fertilized egg. ECPs have no medical contraindications and no known serious medical complications.^6^

**TABLE 1.** Facts About Levonorgestrel-Based Emergency Contraception

**Characteristic**	**Fact**
Dose and regimen	1.5 mg taken at one time, within 120 hours of unprotected sex, is the best approach.
Mechanism of action	Preventing/delaying/disrupting ovulation.
	Possibly thickening cervical mucus.
	**Does not prevent implantation based on best evidence.**
	**Does not disrupt established pregnancy.**
Effectiveness	52%–94% reduction in what risk of pregnancy would have been; better if taken sooner after unprotected sex.
Medical eligibility	Should not be taken if the woman has a confirmed pregnancy (because no need to take), although best evidence indicates it would not harm fetus.
	**Otherwise, no medical restrictions, including age.**
Side effects	Minimal and not harmful (for example, possible mild nausea, menstrual changes).
Repeat use	Regular repeat use not recommended because of relatively poor effectiveness over time and possible side effects such as menstrual irregularity. However, **repeat use poses no known health risks.**

Adapted from the World Health Organization.^5^

Emergency contraceptive pills work primarily by preventing ovulation.

In 2002, the Ministry of Health and Family Welfare (MoHFW) introduced ECPs in the family welfare program. Despite opposition from some medical doctors, moral activists, and parents' associations, in 2005, the method was introduced as an over-the-counter (OTC) drug.

Even with these supportive programmatic changes, evidence shows that awareness and use of ECPs is extremely low. National Demographic and Health Survey data show that less than 1% of married women (0.5% in rural areas and 0.8% in urban areas) have ever used ECPs, and less than one-third of unmarried women know about ECPs.^2^ However, according to a supply audit by AC Nielsen ORG-MARG Research Ltd., a market research company in India, use of ECPs in large metro cities has become substantial. For example, in 2010, 15.2 million ECPs were sold in India, of which 10.4 million, or 68%, were sold in urban areas where only 28% of the country's population lives. In comparison, 4.8 million ECPs, or 32%, were sold in rural areas where 72% of the country's population lives. The largest 15 metropolitan cities, where 22% of the urban population lives, accounted for 3.7 million pills (36% of urban sales), indicating that even in urban areas, access to and use of ECPs are skewed toward large metropolitan cities.

Emergency contraceptive pills have been available over-the-counter in India since 2005, but awareness and use of the method remain low.

Some of the main barriers to mainstreaming use of ECPs come from reservations among physicians and professional associations, who oppose ECPs as an OTC drug. Statements made in national newspapers reflect physicians' strong reservations against easy ECP access and OTC sales. However, these opinions are based on inadequate knowledge and misconceptions about side effects and the mechanism of action of ECPs.^7^^–^^10^ For example, an article published in a national newspaper with high circulation in India stated:

*… the brazen abuse of the OTC emergency drug is triggering severe side-effects, and sometimes even failing to prevent pregnancy, forcing girls to suffer the agony of successive abortions*.^11^

Similarly, a well-read popular magazine, *India Today,* quoted a leading gynecologist saying:

… *cases of herpes, HIV and hepatitis have increased over the past few years because people are increasingly replacing the condom with the emergency pill.*^12^

Another gynecologist said:

*With the influence of Western culture and values, young people view casual sex as a normal activity, making the pill a powerful tool in a woman's hands.*^12^

Such publicly expressed attitudes create medical barriers—provider practices derived partly from a medical rationale but that result in a scientifically unjustifiable barrier or denial of contraception.^13^ Providers at public and private facilities, particularly self-identified “moral police” and activists, contribute to further corrupting the opinions of the community. Some doctors, however, do believe that access to and use of ECPs will reduce the abortion rate.^14^

Some physicians also oppose provision and distribution of ECPs by paramedics or community health workers (CHWs). However, studies show that CHWs can easily provide quality ECP services.^10^ In India, ASHAs (accredited social health activists), a CHW cadre paid based on performance of certain activities, have become an integral part of the health delivery system. There are currently 900,000 ASHAs throughout the country. Under a new scheme, ASHAs are the depot holder of condoms, oral contraceptive pills, and ECPs.^15^ Studies, however, show that ECP awareness among ASHAs is low (15%).^16^ In addition, government facilities have inadequate supplies of ECPs and frequent stockouts, and, in a few cases, some facilities report never having received any ECP supply.^16^^,^^17^

Medical barriers to ECPs, especially as an OTC product, have been reported globally. Often those opposing OTC sales lack knowledge about the mechanism of action and have a misunderstanding that ECPs induce abortion, or they fear that ECPs may be used for regular contraception and misused by adolescents.^16^^–^^20^ Parents also fear that wide availability of ECPs will increase promiscuous, premarital, and irresponsible sex as well as increase the risk of acquiring sexually transmitted infections (STIs).^18^^–^^22^

This article explores the perceptions and attitudes of medical doctors in India about ECPs and how those might contribute to medical barriers and reduced access.[Fig f01]

**Figure f01:**
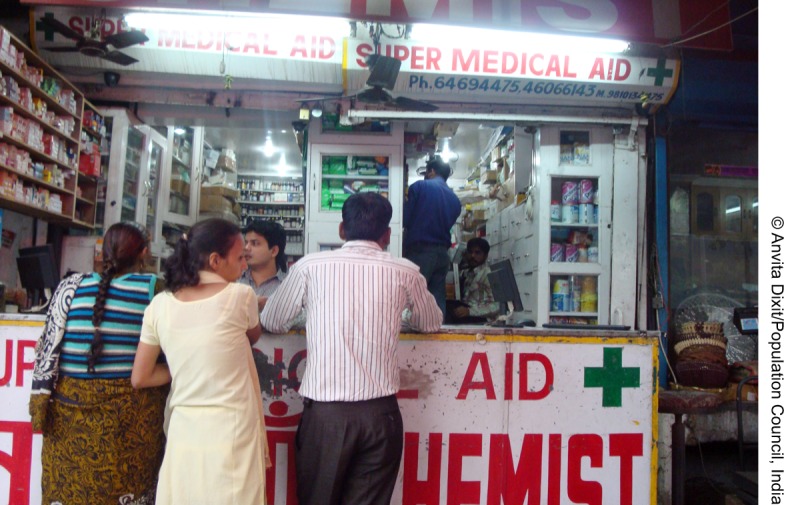
In 2005, India passed legislation allowing over-the-counter sales of emergency contraceptive pills (ECPs), such as at this chemist shop located in New Delhi. Awareness and use of ECPs, however, remain low in the country.

## METHODS

In 2011, we interviewed 83 medical doctors (63 gynecologists and 20 general practitioners or other specialists) who provide ECP information or services using a pretested structured questionnaire.

A list of doctors prepared by the Urban Health Initiative, a family planning initiative funded by the Bill and Melinda Gates Foundation, was used as the sampling frame to select participants. Doctors were randomly selected from 3 large cities in Uttar Pradesh (UP)—Agra, Aligarh, and Lucknow. The state of UP is the largest and most populous state (with 199 million residents) in India, with one of the highest maternal mortality rates, low contraceptive use, and high rates of unmet family planning need and unintended pregnancy.

To complement the quantitative survey data, 19 key opinion leaders were interviewed in depth. Of these, 8 are well-regarded, influential, senior gynecologists.

## RESULTS

### Profile of the Doctors

Almost all the doctors interviewed were Hindu (99%) and female (98%), aged between 24 and 66 years (mean  =  43.3, standard deviation  =  8.4). The majority (78%) was from the private sector. On average, they had been working for 16 years, and 66% had a Doctor of Medicine degree. Most (68%) had received training in family planning, and 43% had received some orientation or training on ECPs.

### Knowledge of Mechanism of Action and Contraindications

Most of the doctors (96%) believed that ECPs work by preventing implantation (Table 2). The latest literature indicates that ECPs do not prevent implantation in any way and that they do not cause any harm to a fertilized egg.^6^ However, at the time of this study, the 20^th^ edition of *Contraceptive Technology*,^27^ published in 2011, reported the mechanism of action as disrupting or delaying ovulation or preventing implantation of a fertilized egg.

**TABLE 2.** Knowledge and Attitudes About ECPs Among Surveyed Doctors in North India (N = 83)

**Knowledge and Attitudes**	**Percentage**
Knowledge of Mechanism of Action^a^	
Inhibits ovulation^b^	23
Prevents implantation	96
Induces abortion	1
Attitudes About Expanding Access^a^	
Oppose OTC provision	67
Oppose provision by CHWs	53
Oppose provision as prophylactic	42
Recommend age restriction	84
Women Using ECPs Are More Likely To^a^:	
Engage in premarital sex	53
Engage in promiscuity/have more sex partners	75
Participate in risky sex behavior	18
Not use other family planning methods	33
Have sexually transmitted infections	26

Abbreviations: CHWs, community health workers; ECPs, emergency contraceptive pills; OTC, over-the-counter.

a Multiple responses were possible.

b Correct response.

Although only one doctor reported the mechanism as inducing abortion, after direct probing on whether ECPs could induce abortion, 10% replied positively. This indicates that the mechanism of action of ECPs is still not clear to many doctors, even senior gynecologists. Reinforcing these observations, a senior gynecologist working in public hospital said:

*It is a long-acting progesterone. It hinders in the implantation; it makes the endometrium hostile for the fertilized ovum*.

Yet another gynecologist working in a large private hospital said:

*ECP doesn't allow implantation of the zygote. I do not think people have any confusion regarding this*.

All doctors had correct knowledge about the number of ECPs that need to be taken, at what interval (in the case of the combined 2-pill regimen), and the number of hours within which ECPs must be taken after unprotected sex to be effective in preventing pregnancy. Most doctors (88%) believed that ECPs are safe, with some side effects.

All surveyed doctors knew the correct dose and regimen for emergency contraceptive pills, but there was confusion around contraindications.

However, there was confusion around contraindications; many believed that use of ECPs could be harmful if taken by pregnant women (59%), by patients with heart disease (37%) or liver disease (46%), or by women who are breastfeeding (27%). Only 3 doctors correctly answered that there are no contraindications to ECPs, and only 16% were aware of the national guidelines on ECPs.

### Attitudes About ECP Provision

Even though 88% of doctors believed that ECPs are safe, two-thirds of the doctors were against providing ECPs as an OTC drug (Table 2).

The Head of the Obstetrics and Gynecology Department of a renowned medical college said:

I personally feel that it should not be sold as an OTC drug. Though it reduced unwanted pregnancy, misuse is increasing. I feel that publicity of ECPs and its availability as an OTC product is not good. Women come to us only when complications arise due to repeat use; [they] never come for counseling or advice before use. The main reason for such misuse is the lack of knowledge and awareness among the public regarding its appropriate use.

A chief medical superintendent at a district hospital, expressing his reservations of making ECPs available OTC, said:

The central government needs to ensure that ECPs are prescribed only at hospitals by the doctors. Neither the abortion pills nor the ECPs should be OTC, because girls are misusing them.

More than half of the doctors also had reservations about easy access to ECPs, through paramedics or CHWs, and 42% opposed providing the method prophylactically (Table 2). Many respondents were concerned that paramedics and CHWs would potentially “misuse” (50%) the method and that they would have poor knowledge about ECPs (32%). A senior doctor said:

*None of the ANMs [auxiliary nurse-midwives] or CHWs have got any training on ECPs; they learnt by practice and by working with us. Even doctors have not been trained*. *Then how will anyone know about the exact or the appropriate use of ECPs?*

Most of the doctors (84%) wanted a minimum age restriction for obtaining ECPs. The age restriction ranged from 16–25 years, with the majority (54%) in favor of 18–22 years. Most (95%) did not think programs to increase ECP accessibility were necessary because they believed ECPs were already easily available from shops. However, shop audit reports from AC Nielsen ORG-MARG Research Ltd., which provides some of the most authentic data on pharmaceutical industries, show very limited ECP stocks in rural areas, probably reflecting the general lack of knowledge about ECPs among people, and thus the very low uptake.^2^^,^^28^

Most surveyed doctors wanted to impose age restrictions on use of ECPs.

### Attitudes About ECP Users

Survey respondents generally had negative moral judgments about ECP users, which probably also negatively impacts access to ECPs. For example, 53% of respondents thought that women using ECPs are more likely to have premarital sex, and 75% thought they would have more sex partners (Table 2).

Surveyed doctors generally had negative attitudes about ECP users.

These attitudes were also reflected in the in-depth interviews. For example, a gynecologist from a private clinic expressed:

I do feel that the easy availability of ECPs has increased the sexual contacts among young girls. There is a lot of misuse of ECPs by the current generation.

### Perceptions of When to Use ECPs

In India, societal norms prohibit people from openly condoning use of ECPs by adolescents. However, almost two-thirds of the doctors thought that it was appropriate for women having infrequent sex to use ECPs as a family planning method, potentially reflecting their openness to mainstreaming ECPs (Table 3).

**TABLE 3.** Attitudes of Surveyed Doctors About Appropriate Use of ECPs (N = 83)

**Situation/Characteristic of User**	**Percentage**
Married woman	80
Infrequent/unpredictable sex	61
Contraceptive failure	12
Unprotected sex	41
Rape or sexual coercion	27
Living in refugee/conflict settings	7
Multiple responses were possible.

Most surveyed doctors thought it was appropriate for women having infrequent sex to use ECPs.

Interestingly, more than three-fourths of the doctors thought that being married was an important criterion for appropriate use of ECPs, which might be more of a reflection of social norms than bias among the doctors. A senior gynecologist explained:

*I think unmarried girls who ar*e *exploring their sexuality and having infrequent sex with their boyfriends can use it.*

Most doctors felt that women under 29 years of age (68%) and married women (68%) were currently the main ECP users while only 21% of the doctors believed that unmarried young women used ECPs.

Two-thirds of the doctors interviewed believed there were no barriers in accessing or using ECPs. However, about 5% felt that there could be low demand for ECPs because of lack of information. A gynecologist from a public health facility reported:

… *demand for ECPs is also very low. One year back, I received a box of ECPs. Some of them are still in stock. Soon all of them will expire. People who live in the vicinity of the clinic are poor and are not educated. They do not know much about the method.*

### Repeat Use and Perceptions of Side Effects

There was substantial confusion about the definition of repeat use and about its possible adverse consequences. The initial reaction of 78% of the doctors interviewed was that ECPs should not be used more than once in one menstrual cycle because it is not a regular method of family planning: “*It is an emergency pill.*”

A senior gynecologist from a public hospital said:

If a woman is taking ECPs several times, it means she is using it as a regular contraceptive. She needs to understand its harmful effect … ECPs are for [a] real emergency.

This is in line with national guidelines. Only 15 doctors (18%) felt that ECPs could be used more than once within the same menstrual cycle. Of these, 8 doctors suggested ECPs could be used up to 2 times, while the remaining 7 felt that it could be used more than 2 times, depending on the need.

Doctors' perceptions of the possible side effects of repeat use varied, from menstrual disturbance/irregular bleeding, including excessive bleeding during menstruation (85%), to vomiting/nausea (20%) and weakness (12%). About 24% held the misperception that ECPs could cause ectopic pregnancy and infertility.

During in-depth interviews, many senior gynecologists expressed concerns regarding a high failure rate and possibility of ectopic pregnancy:

*My colleagues have concerns about repeat use of ECPs. They question it because of [the] misconception of the increased chance of ectopic pregnancy and extent of failure rate. We do not have answers to such questions*.

A substantial proportion (27%) of the 63 surveyed gynecologists reported that in the last month they were presented with 1 to 3 cases of pregnancy due to ECP failure. Based on their personal experiences, 40% of the gynecologists reported that there is an increase in patients with menstrual problems after using ECPs.

A senior gynecologist from a private hospital said:

There should be a rider on [the] ECP pack that the client should follow up with a gynecologist within 15 days of taking it, and then we can counsel women on the failure rates. Often the user has a little bleeding and confuses it as a normal period. But actually they have missed their period and when they come to us, they come with an advanced pregnancy.

### Perceptions of Reduction in Abortions

Most gynecologists felt that there is little or no evidence to suggest that the abortion-seeking practice among young and unmarried girls had changed as a result of having ECPs available. In contrast, 5 gynecologists (8%) said that they were experiencing an increase in the number of induced abortions due to failure of ECPs. However, during in-depth interviews, 3 gynecologists reported that the frequency of very young girls seeking abortion had decreased. In the words of a senior gynecologist from Lucknow:

… the number of young girls who used to come to me for abortion has declined. In my own clinic, 2–3 young girls used to come every month. Now it is rare.

### Recommendations for Mainstreaming ECPs

Two-thirds of the doctors surveyed recommended educational campaigns to disseminate information about ECPs and its correct use; they felt that public knowledge is negligible except among the educated urban class. A senior gynecologist from a public health hospital explained:

[Initially] ECP was being advertised as something very attractive to use. But it is not like that. The way we create awareness, educate, and counsel the public and the providers is very important. It should be done in such a way that it is something that should be used only in accidents or emergency. It should not be advertised as a contraceptive method and people should know it is much less effective if used frequently as a regular method.

The 2 main modes of communication that were recommended included advertising (100%), using mass and mid-media, and raising awareness through CHWs and other providers (67%), including doctors. A few (6%) felt that there was no need for any such initiatives, as they would lead to misuse.

On the topic of affordability, about one-third of the doctors did not feel a need to reduce costs or to increase free distribution of ECPs at public clinics. A gynecologist suggested:

Getting ECPs is not a problem as it is an OTC drug. You can go to an unknown chemist to buy ECPs, if you want privacy. Even the cost [ranging from Rs. 40 to Rs. 100] is not much.

## DISCUSSION

This study shows that many physicians in North India lack up-to-date information on the mechanism of action of ECPs. But this may be because the study was carried out at the end of 2011, while information on mechanism of action was revised and updated in 2012. Some doctors have the misconception that the mechanism of action of ECPs could lead to abortions. Many of them also have misconceptions about contraindications.

Doctors' biases reported in earlier literature were confirmed by this study.^18^ A majority of doctors oppose ECPs as a prophylactic and as an OTC drug. They also strongly believe that there should be an age restriction on the provision of ECPs, due to their experience with failure and side effects. Further, they believe that “easy” availability of ECPs would promote premarital sex and promiscuity, replace regular family planning methods, and increase chances of acquiring STIs through “irresponsible” sexual behavior. Similar opinions and concerns were expressed at 2 national consultations on emergency contraception organized by the Population Council and held in Delhi and Mumbai, which were attended by more than 100 national and international representatives, including from the Indian Medical Association (IMA), the Federation of Obstetric and Gynaecological Societies of India (FOGSI), the MoHFW, researchers, and ECP manufacturers.

Although ECPs are not recommended for use as a regular contraceptive because overall effectiveness is reduced over time, repeat use poses no known health risks.^5^ Furthermore, there is no research to limit the number of times that ECPs can be used within a menstrual cycle.^6^ Many gynecologists, however, expressed a fear of repeat use of ECPs and thought that more patients were coming to them with pregnancy due to repeat use of ECPs. Although failure of ECPs might be related to its repeated use, it could also be a factor of the interval between unprotected sex and ECP use or to the stage of the menstrual cycle when ECPs are taken. Educational campaigns for both providers and users are needed to address recommendations on repeat use of ECPs.

A few of the interviewed gynecologists feel that abortions have decreased since ECPs have become available. However, studies suggest there is no public health impact on abortion rates of ECP use.^29^^,^^30^ This gap in knowledge among doctors in India needs to be addressed. At the same time, the perception of reduced numbers of abortions, while it cannot be generalized or attributed to ECP use, should be explored further, for example, through a prospective study to assess whether easy access to ECPs will decrease unwanted pregnancies and the need for abortion.

In general, negative attitudes of physicians toward ECPs constitute significant medical barriers to mainstreaming the method into national programs as physicians are important opinion leaders. As doctors and their professional associations exert strong influence on policymaking, misconceptions and provider biases could be serious constraints or could delay decisions that could make ECP access easier at an affordable price.

Negative attitudes of physicians pose significant medical barriers to mainstreaming emergency contraception into national programs.

To overcome these obstacles, evidence-based advocacy to address doctors' reservations and a sustained educational campaign to disseminate correct information about ECPs to all stakeholders, including opinion leaders, providers, and the community at large, are needed. Support and positive advocacy by medical associations such as the IMA and FOGSI could be effective in reducing providers' biases and medical barriers. Interventions that could potentially reduce medical barriers among doctors include lectures on ECPs in the final year of medical students' training and half-day ECP orientation as part of on-the-job training. These activities could be done at very low cost and completed within 4 to 6 months.

Our recent efforts with FOGSI helped to increase discussions about ECPs. Information about ECPs, including benefits and mechanism of action, has been disseminated to each chapter of the association, and at least one session on ECPs is being organized at the annual conference. Efforts are required to make this a regular practice, for example, through continual dissemination of accurate information about ECPs such as through the FOGSI newsletter and journal. The key idea is to make the environment conducive for correct knowledge about ECPs, the role of the method in the national Family Welfare Program, and to make the method easily available and accessible to all.

One recent positive step in the direction toward increasing knowledge and reducing negative attitudes is the withdrawal of the government ban on ECP advertisement, so that ECP manufacturers can also play a supportive role in addressing the barriers and negative attitudes toward ECP use. To conclude, the study suggests an urgent need for advocacy among doctors and other stakeholders to make ECPs available to reduce unintended pregnancy.

### Limitations

There are a few limitations to the study. First, it had a small sample size and was concentrated largely in one state, although it is one of the largest states in India and one with high unmet need. Second, the study was carried out in 2011, but updated guidelines on mechanism of action of ECPs were published in 2012, so we are unable to say confidently whether the doctors had correct knowledge about this critical piece of information.
